# A Prediction Rule for Overall Survival in Non-Small-Cell Lung Cancer Patients with a Pathological Tumor Size Less Than 30 mm

**DOI:** 10.1155/2019/8435893

**Published:** 2019-05-02

**Authors:** Wang-Yu Zhu, Ke-xin Fang, Jian-ying He, Ri Cui, Yong-Kui Zhang, Han-bo Le

**Affiliations:** ^1^Cell and Molecular Biology Laboratory, Zhoushan Hospital, Wenzhou Medical University, Zhoushan, Zhejiang Province, China; ^2^Department of Cardiothoracic Surgery, Zhoushan Hospital, Wenzhou Medical University, Zhoushan, Zhejiang Province, China; ^3^Lung Cancer Research Center, Zhoushan Hospital, Wenzhou Medical University, Zhoushan, Zhejiang Province, China; ^4^Affiliated Zhoushan Hospital and School of Pharmaceutical Sciences, Wenzhou Medical University, Wenzhou, Zhejiang 325035, China

## Abstract

We sought to develop and validate a clinical nomogram model for predicting overall survival (OS) in non-small-cell lung cancer (NSCLC) patients with resected tumors that were 30 mm or smaller, using clinical data and molecular marker findings. We retrospectively analyzed 786 NSCLC patients with a pathological tumor size less than 30 mm who underwent surgery between 2007 and 2017 at our institution. We identified and integrated significant prognostic factors to build the nomogram model using the training set, which was subjected to the internal data validation. The prognostic performance was calibrated and evaluated by the concordance index (C-index) and risk group stratification. Multivariable analysis identified the pathological tumor size, lymph node metastasis, and Ki-67 expression as independent prognostic factors, which were entered into the nomogram model. The nomogram-predicted probabilities of OS at 1 year, 3 years, and 5 years posttreatment represented optimal concordance with the actual observations. Harrell's C-index of the constructed nomogram with the training set was 0.856 (95% CI: 0.804-0.908), whereas TNM staging was 0.814 (95% CI: 0.742-0.886, *P* = 5.280221*e* − 13). Survival analysis demonstrated that NSCLC subgroups showed significant differences in the training and validation sets (*P* < 0.001). A nomogram model was established for predicting survival in NSCLC patients with a pathological tumor size less than 30 mm, which would be further validated using demographic and clinicopathological data. In the future, this prognostic model may assist clinicians during treatment planning and clinical studies.

## 1. Introduction

Despite significant treatment advancements, lung cancer remains the leading cause of cancer-related mortality worldwide with non-small-cell lung cancer (NSCLC) accounting for 85% of all lung cancer cases [1, 2]. Currently, lung adenocarcinoma and squamous cell lung cancer (SCC) are the two most commonly diagnosed forms of NSCLC. Due to the use of low-dose computed tomography (LDCT) in high-risk and some healthy subjects, it has become easier to detect the disease during its early stages when treatment is most effective [3]. Despite dramatic improvements in diagnosing lung cancer, the 5-year cumulative survival rate for NSCLC has remained unchanged at 18.5%. However, most studies have assessed the overall survival (OS) in patients with advanced-stage NSCLC, as only a limited number of patients were diagnosed with the early-stage disease in the past [4]. Nevertheless, some patients with the early-stage NSCLC present with aggressive characteristics, and there is limited information on how to estimate the survival of these patients. Currently, a limited number of studies have used mathematical models to predict the survival outcomes of patients with early-stage NSCLC [5, 6]. The development of prognostic models may aid clinicians during treatment planning and patient stratification in the future.

While several prognostic biomarkers have been investigated in lung cancer, there have been limited imaging agents that have advanced to clinical trials. For example, preoperational or initial peripheral blood carcinoembryonic antigen (CEA) levels were previously shown to be useful prognostic biomarkers for NSCLC patients [7, 8]. In addition, some immunohistochemical (IHC) markers, such as p53 and Ki-67, have been successfully used for predicting the prognosis of NSCLC patients [9, 10]. Patients with a mutated epidermal growth factor receptor (EGFR) were also shown to benefit from specific molecular-targeted therapies [11]. However, the prognostic role of EGFR-targeted agents in NSCLC patients with a pathological tumor size less than 30 mm remains unclear. The new substaging system defined in the 8th edition of the American Joint Committee on Cancer (AJCC) divides stage IA into IA1, IA2, and IA3, which has shown a significant prognostic value for patients with NSCLC [12]. In addition, other prognostic factors may be used in NSCLC patients with a pathological tumor size less than 30 mm, such as smoking status, histopathology subtype, and lymph node metastasis [13]. The combined prognostic factors based on a cohort may aid in the precise assessment of the disease prognosis in NSCLC patients. Recently, several studies have shown that nomogram models can be superior to the traditional TNM staging system for the prediction of patient outcomes in several types of cancer [14–16]. Nomograms can be used to present an intuitive graph of the results from the statistical predictive model, which makes it possible to quantify the prognostic probability for predicting clinical events individually for each patient.

Therefore, the goal of this study was to develop and validate an available nomogram model by combining clinicopathological variables and molecular biomarkers based on the data obtained from NSCLC patients with a pathological tumor size less than 30 mm from the eastern islands of China. We also sought to compare the prognostic value of a nomogram model with the newest TNM staging system.

## 2. Material and Methods

### 2.1. Patient Population

Data were collected from patients treated in the Lung Cancer Research Center of Zhoushan Hospital, Zhejiang Province, China, from January 2007 to December 2017. Since 2007, all patients who underwent surgery with a pathological diagnosis of primary lung cancer were in the database and contacted for follow-up. In total, 786 of the 2434 patients in the database met the inclusion criteria for this study. Tumors that were histologically classified prior to 2011 were reassessed and classified by two senior pathologists in accordance with the World Health Organization Classification and Lung Adenocarcinoma Subtypes followed by the New International Association for the Study of Lung Cancer and the American Thoracic Society and European Respiratory Society (IASLC/ATS/ERS) [17]. The staging was determined following the new substage guidelines found in the 8th edition of the AJCC [12].

Demographic data, including age, sex, history of tobacco exposure, and pathology, which includes histological type, pathological tumor size, lymph node metastasis, tumor location, and pleural invasion, were obtained. In addition, the type of surgical intervention and pathological TNM stage were included. Other factors, such as preoperational peripheral CEA, IHC markers for p53 and Ki-67 expression, and EGFR mutations, were also included. Follow-up data were obtained from the death registration system of the Zhoushan Center for Disease Control and Prevention, along with the medical review of all patients on an outpatient basis with computed tomography (CT) imaging at 3-month intervals for the first year after treatment and then at 6-month intervals.

The exclusion criteria for this study included patients with tumor sizes greater than 30 mm in diameter, small-cell lung cancer (SCLC) cases, large-cell lung cancer (LCLC) cases, lymphoepithelioma-like carcinoma, and neuroendocrine tumors that were 30 mm in diameter or less. Finally, 786 patients were identified to be in this study cohort and separated into the training or validation sets according to their date of surgery. The 457 patients who underwent surgery from 2007 to 2014 were assigned to the training group and used to develop the nomogram prognostic model, while the other 329 patients who underwent surgery from 2015 to 2017 were used to validate the nomogram model. The last follow-up was the date of death or until April 30, 2018, for patients who are still alive. The OS was calculated from the time of surgery until the time of death or the final follow-up. This study was approved by the Ethical Review Committee of Zhoushan Hospital, Zhejiang Province, China. Written content was waived by the Institutional Review Board due to the retrospective nature of this study.

### 2.2. Statistical Analysis

Continuous data were reported as median values with interquartile range. Cumulative survival curves were depicted using the Kaplan-Meier method with a calculated median survival time and a 95% confidence interval (CI). The log-rank test was used to compare the prognostic factors, and the univariate analysis was used to calculate the *P* values. Those *P* values of ≤0.05 were considered statistically significant and used in the multivariate analysis for the Cox proportional hazards regression model. The development of a nomogram for the training set was constructed based on the results of the multivariate analysis using the backward stepwise selection method with the Akaike information criterion (AIC) [18]. The nomogram model was subjected to the internal data validation, and the concordance index (C-index) was calculated to evaluate the predictive accuracy of OS. A larger C-index indicated a more accurate probability to distinguish the outcome of the model. The calibration was estimated using a calibration curve for 1-year, 3-year, and 5-year OS after bias correction.

All statistical analyses were performed using SPSS 22.0 (IBM, Chicago, IL, USA) and R version 3.5.1 (R Foundation for Statistical Computing, Vienna, Austria). *P* values that were ≤0.05 were considered statistically significant with a two-sided test.

## 3. Results

### 3.1. Characteristics of Patients in the Training and Validation Cohorts

A total of 786 cases were identified as having NSCLC with a pathological tumor size less than 30 mm and were separated into the training set (*n* = 457) and validation set (*n* = 329) based on the surgical date. The demographic data and clinicopathological characteristics of the two groups are summarized in Table 1. Among the variables, preoperative serum CEA levels, Ki-67 expression levels, p53 expression levels, and EGFR mutation information were missing in 0.2%, 37.2%, 37.2%, and 41.4% of the cases in the training set and 0.3%, 14.6%, 14.0%, and 0.6% of the cases in the validation set, respectively. The median follow-up intervals were 49 months (range 3-136 months) for the training set and 31 months (range 6-39 months) for the validation set.

### 3.2. Univariate and Multivariate Analyses of the OS in the Training Set

The findings from the univariate and multivariate analyses of OS are described in Table 2. The univariate analysis indicated that patients who were female (vs. male, *P* < 0.001), less than 60 years of age (vs. ≥60 years of age, *P* = 0.004), and nonsmokers (vs. current smokers or those with history of smoking, *P* < 0.001) and had preoperative CEA levels of less than 5.0 ng/mL (vs. ≥5.0, *P* < 0.001) showed better OS.

In histology, patients with NSCLC-subtype adenocarcinoma in situ (AIS) and minimally invasive adenocarcinoma (MIA) had more favorable OS than those patients with IAC or SCC (*P* < 0.001). A pathological tumor size ≤ 10 mm displayed the most favorable OS, followed by a pathological tumor size of 10-20 mm, while larger tumors (20-30 mm) showed the least favorable OS (*P* < 0.001). Patients with no lymph node metastasis showed superior survival than those patients with lymph node metastasis (N1 or N2, *P* < 0.001). For the pathological TNM stage, patients with early-stage disease showed better OS than those with advanced-stage disease (*P* < 0.001). Moreover, patients positive for P53 or Ki-67 expression experienced less favorable OS when compared with those patients negative for P53 (*P* < 0.001) and Ki-67 (*P* < 0.001). However, the surgical procedure had no significant impact on OS between the two groups (*P* = 0.850), as well as EGFR mutation status. However, patients with a mutant EGFR showed worse OS than patients with the wild-type EGFR, yet this finding was not statistically significant (*P* = 0.083). All significant factors identified as predictors of OS in the univariate analysis were used for the multivariate analysis based on the Cox proportional hazards regression. The results described that pathological tumor size (*P* < 0.001), lymph node metastasis (*P* < 0.001), and Ki-67 expression (*P* < 0.001) were the independent prognostic factors in the Cox model.

### 3.3. Development of a Nomogram Model for OS

**Figure 1:**
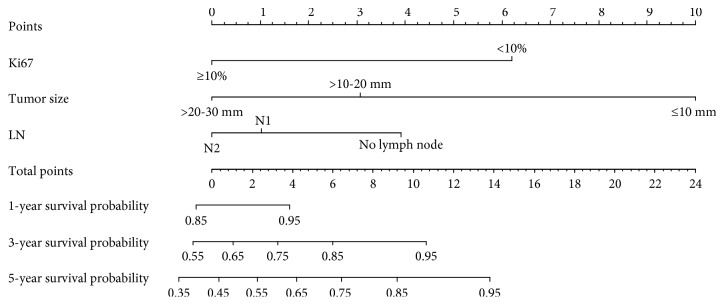
The prognostic nomogram for NSCLC patients with a pathological tumor size of 30 mm or smaller in the training set. Tumor size: pathological tumor size; LN: lymph node metastasis.

The nomogram model was established using the independent significant prognostic factors (Figure 1). The nomogram illustrated the points of each predictor ranging from 0 to 100. The results showed that pathological tumor size was the most significant contributor to the prognosis, followed by Ki-67 expression levels and lymph node metastasis. The total scores were calculated and located on the total point scale. The probabilities of OS at 1 year, 3 years, and 5 years posttreatment were individually estimated by drawing a straight line and ranged from 0.80 to 0.98, 0.50 to 0.95, and 0.35 to 0.95, respectively.

### 3.4. Calibration and Validation of the Nomogram in the Validation Set

**Figure 2:**
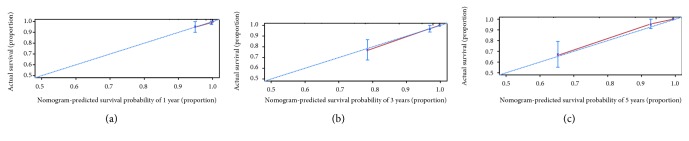
Calibration curves for the prediction of overall survival (OS) in NSCLC patients with a pathological tumor size of 30 mm or smaller in the training cohort. The *x*-axis represents the nomogram-predicted patient survival, while the *y*-axis represents the observed OS at (a) 1 year, (b) 3 years, and (c) 5 years.

**Figure 3:**
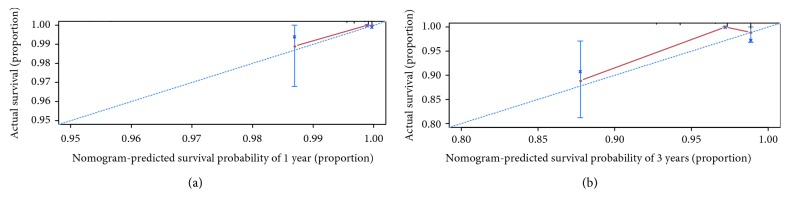
Calibration curves for the prediction of overall survival (OS) in NSCLC patients with a pathological tumor size of 30 mm or smaller in the validation cohort. The *x*-axis represents the nomogram-predicted patient survival, while the *y*-axis represents the observed OS at (a) 1 year and (b) 3 years.

The calibration plot presented an optimal prediction for 1-year, 3-year, and 5-year OS between the nomogram prediction and actual observations (Figure 2). In the validation cohort, the calibration curve also showed an accordant agreement for 1 year and 3 years OS (Figure 3). Harrell's C-index, which was used to evaluate the performance of the constructed nomogram, was 0.856 (95% CI: 0.804-0.908) in the training set and 0.820 in the validation set (95% CI: 0.647-0.993). The TNM staging was 0.814 (95% CI: 0.742-0.886, *P* = 5.280221*e* − 13) in the training set and 0.812 (95% CI: 0.711-0.913, *P* = 0.675) in the validation set.

### 3.5. Stratifying the Risk Ability of the Prognostic Nomogram Model

**Figure 4:**
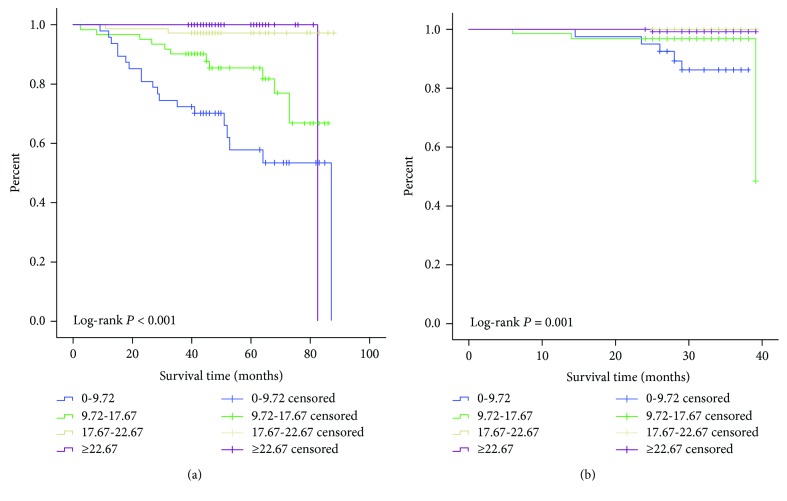
Kaplan-Meier curves of overall survival (OS) for the (a) training set and (b) validation set in T1 NSCLC patients separated by nomogram scores.

We divided patients into four risk groups (scores: 0-9.72, 9.72-17.67, 17.67-22.67, and ≥22.67) with the optimal cut-off values for total points in the training set (Table 3). A survival analysis demonstrated that the subgroups showed significant distinctions within the training cohort (*P* < 0.001, Figure 4(a) and Table 4). The same cut-off values were also applied to the validation set, and survival differences were represented among the subgroups (*P* < 0.001, Figure 4(a) and Table 4).

## 4. Discussion

In recent years, the increased usage of CT screening has led to an increase in the number of lung cancers with a pathological tumor size less than 30 mm being detected in the clinic [19, 20]. However, the prognostic prediction capabilities of a nomogram model have not been constructed for NSCLC patients with a pathological tumor size less than 30 mm. In this study, we developed a nomogram model and internally validated it to predict the prognosis of NSCLC patients from a single institution in the eastern islands of China. This nomogram was not only based on demographic data and clinicopathological characteristics but also focused on the molecular factors and IHC markers. We proposed that the nomogram could allow for better treatment planning in the future.

A nomogram model established on the data from multiple institutions often yields higher accuracy and less bias. However, the nomogram model in this study used data derived from a single institution. In addition, molecular marker data were included in this study. As a vital tumor suppressor, p53 expression is often lost in tumors, which can be directly correlated with the prognosis of patients [21]. Ki-67 is a marker of cell proliferation in NSCLC, and elevated Ki-67 levels have been correlated with poor outcomes in NSCLC patients [9]. Moreover, an *EGFR* mutation was previously discovered to have a prognostic role in NSCLC patients. Our univariate analysis revealed that patients positive for p53 or Ki-67 expression showed less favorable outcomes than those patients who were negative for p53 or Ki-67 in the training cohort. However, the *EGFR* mutation status had no significant effect on the outcome of NSCLC patients. In general, these findings agreed with the results from other studies [10, 22]. In addition, sex, age, smoking status, preoperational CEA levels, histology, pathological T categories, lymph node metastasis, and pathological TNM stage were also found to be prognostic factors in the univariable analysis. Through the subsequent multivariable analysis, the pathological T stage and N category, as well as Ki-67 expression, were identified as independent prognostic predictors. Previous studies also demonstrated that tumor size and lymph node metastasis were risk factors for NSCLC [4, 23]. Notably, Ki-67 expression was found to be associated with poorer survival outcomes in NSCLC patients. To our knowledge, this is the first attempt to include an IHC marker into a nomogram model.

The nomogram model showed a clear distinction capacity for predicting patient outcomes in the training cohort. The C-index of this model was 0.856, higher than those previously reported in published studies [23, 24]. Moreover, the nomogram model was more successfully applied than the AJCC TNM staging classification system in the individual evaluation of patient prognosis, which may be attributed to the inclusion of Ki-67 expression data. IHC markers can provide valuable insight into the pathology of NSCLC, and molecular markers are commonly used in the pathological diagnosis of lung cancer in the clinic [25]. We used molecular markers to build our nomogram model, aiming to increase its overall accuracy. In addition, a relatively good calibration was observed in the nomogram of the training cohort. To validate the nomogram model, we use internal validation data to evaluate the accuracy and calibrate the model. The C-index reflected a good discrimination power in the validation data, but it was lower than that of the training set. This might be due to the shorter follow-up times for patients in the validation cohort. Moreover, using an optimal cut-off value, the nomogram showed excellent prediction capabilities in terms of OS in different risk subgroups. The proposed nomogram may have a potential role in clinically evaluating the OS probability of patients with NSCLC [26].

There were several limitations to this study. The first limitation was the amount of missing data in the training data, such as Ki-67 and p53 expression levels and *EGFR* mutation status. This might introduce selection bias into the nomogram model. The second limitation is that this study was conducted at a single institution and the established nomogram was validated using an internal cohort. External validation based on a larger number of patients at multiple institutions should be introduced in the future. The third limitation was the retrospective nature of this study that had shorter follow-ups, especially in the validation set. Lastly, the fourth limitation was our inability to include some recognized prognostic parameters, such as comorbidity and postoperative complications in the nomogram. Other parameters were not assessed in this study, such as treatment efficacy, disease-free interval, or progression-free survival. In future studies, we will improve the model by using multi-institutional data with longer follow-up times, less missing data, and the presence of other predictive factors.

In the present study, we developed a prognostic nomogram model for NSCLC patients with a pathological tumor size less than 30 mm and validated the model using an internal cohort. We also built proportional OS subgroups in the model to discriminate between different patient outcomes. We developed a high-performance nomogram model that includes molecular marker data and displays a C-index of 0.856. This nomogram could be used as a convenient and precise outcome predictive tool for clinicians in the future, yet further external validations using data from multiple institutions should be considered.

## Figures and Tables

**Table 1:** The overall survival of the training set and validation set in NSCLC patients with a pathological tumor size less than 30 mm.

Patient characteristics	Training set (*n* = 457)				Validation set (*n* = 329)			
	Patients (#)	Patients (%)	OS (months)		Patients (#)	Patients (%)	OS (months)	
			Median	95% CI			Median	95% CI
Sex								
Male	193	42.2	92.7	85.3-100.0	113	34.3	38.0	37.1-38.9
Female	264	57.8	117.4	111.5-123.2	216	65.7	38.8	38.5-39.1
Age (y), median (IQR)	60 (52-66)				57 (51-65)			
<60	223	48.8	114.8	108.4-121.2	186	56.5	38.5	38.0-39.0
≥60	234	51.2	100.8	93.8-107.8	143	43.5	38.5	38.2-38.9
Smoking								
Never smoker	315	68.9	114.1	108.4-119.7	258	78.4	38.8	38.6-39.1
Current/former smoker	142	31.1	91.8	83.4-100.1	71	21.6	37.4	38.6-39.1
Preoperational CEA level (ng/mL), median (IQR)	1.95 (1.28-3.27)	*n* = 456			1.75 (1.14-2.79)	*n* = 328		
<5.0	395	86.6	108.7	103.9-113.4	297	90.5	38.7	38.4-39.0
≥5.0	61	13.4	82.3	68.4-96.2	31	9.5	35.8	33.2-38.3
Type of surgery								
Lobectomy	383	83.8	107.4	102.2-112.5	127	38.6	38.7	38.4-39.1
Limited resection	74	16.2	91.4	78.5-104.3	202	61.4	38.2	37.4-38.9
Tumor location								
Right upper lobe	161	35.2	110.1	102.6-117.6	118	35.9	NA	
Right middle lobe	35	7.7	117.3	102.0-132.6	34	10.3		
Right lower lobe	68	14.9	87.6	75.5-99.6	56	17.0		
Left upper lobe	124	27.1	114.1	104.4-123.9	88	26.8		
Left lower lobe	58	12.7	97.4	85.0-109.8	31	9.4		
Undefined	11	2.4	72.9	43.5-102.2	2	0.6		
Histology								
AIS & MIA	195	42.7	127.6	124.8-130.4	195	59.3	38.9	38.8-39.1
IAC	198	43.3	97.6	89.8-105.4	123	37.4	38.0	37.1-38.9
SCC	64	14.0	73.6	62.4-84.8	11	3.3	33.2	31.7-34.7
Pathological tumor size (mm), median (IQR)	15 (9-20)							
≤10 mm	156	34.1	126.0	121.5-130.5	180	54.7	38.9	38.8-39.1
>10-20 mm	196	42.9	107.3	100.6-114.1	106	32.2	38.3	37.5-39.1
>20-30 mm	105	23.0	81.3	71.1-91.5	43	13.1	37.5	35.8-39.1
Lymph node metastasis								
N0	402	88.0	114.1	109.3-119.0	311	94.5	38.8	38.5-39.1
N1	23	5.0	65.2	45.9-84.6	5	1.5	26.9	21.5-32.3
N2	32	7.0	54.4	62.4-84.8	13	4.0	34.2	31.1-37.3
Pathological TNM stage								
0	111	24.4	128.7	126.1-131.2	99	30.1	NA	
IA1	77	16.8	110.9	100.1-121.7	92	28.0		
IA2	138	30.2	105.5	96.8-114.1	89	27.0		
IA3	77	16.8	91.8	79.7-104.0	31	9.4		
IIB	23	5.0	65.2	45.9-84.6	5	1.5		
IIIA	31	6.8	54.8	42.1-67.5	13	4.0		
P53 expression	*n* = 287				*n* = 281			
Negative	208	72.5	83.3	80.7-85.9	211	75.1	38.8	38.5-39.1
Positive	79	27.5	68.2	61.2-75.2	70	24.9	36.5	35.2-37.7
Ki-67 expression	*n* = 287				*n* = 283			
<10%	203	70.7	84.2	81.4-87.1	186	65.7	38.9	38.7-39.1
≥10%	84	29.3	67.6	61.2-74.0	97	34.3	37.6	36.5-38.7
EGFR mutation	*n* = 268				*n* = 327			
Wild type	190	70.9	76.9	74.8-78.9	182	55.7	38.3	37.7-38.9
Mutation	78	29.1	71.4	64.7-78.0	145	44.3	38.8	38.4-39.2

IQR: interquartile range; CEA: carcinoembryonic antigen; AIS: adenocarcinoma *in situ*; MIA: minimally invasive adenocarcinoma; IAC: invasive adenocarcinoma; SCC: squamous cell lung cancer; EGFR: epidermal growth factor receptor.

**Table 2:** Univariable analysis and Cox proportional hazards regression analysis.

Variable	Univariable analysis			Multivariable analysis		
	Hazard ratio	95% CI	*P*	Hazard ratio	95% CI	*P*
Sex			<0.001^∗^			
Female	Reference					
Male	2.833	1.865 to 4.304				
Age (y), median (IQR)			0.004^∗^			
<60	Reference					
≥60	1.849	1.221 to 2.799				
Smoking status			<0.001^∗^			
Never smoker	Reference					
Current/former smoker	2.394	1.612 to 3.556				
Preoperational CEA level (ng/mL)			<0.001^∗^			
<5.0	Reference					
≥5.0	2.801	1.807 to 4.342				
Type of surgery			0.850			
Lobectomy	Reference					
Limited resection	1.058	0.588 to 1.906				
Histology			<0.001^∗^			
AIS & MIA	Reference					
IAC	21.102	6.614 to 67.326	<0.001^∗^			
SCC	40.946	12.621 to 132.841	<0.001^∗^			
Pathological tumor size (mm)			<0.001^∗^			0.011^∗^
≤10 mm	Reference			Reference		
10-20 mm	10.548	3.278 to 33.947	<0.001^∗^	6.280	0.783 to 50.367	0.084
>20-30 mm	25.350	7.904 to 81.311	<0.001^∗^	13.831	1.711 to 111.823	0.014^∗^
Lymph node metastasis			<0.001^∗^			0.037^∗^
N0	Reference			Reference		
N1	4.689	2.522 to 8.718	<0.001^∗^	2.606	0.964 to 7.049	0.059
N2	6.849	4.270 to 10.985	<0.001^∗^	2.970	1.170 to 7.539	0.022^∗^
Pathological TNM stage			<0.001^∗^			
0	Reference					
IA1	7.007	0.726 to 67.588	0.092			
IA2	30.748	4.192 to 225.536	0.001^∗^			
IA3	53.342	7.260 to 391.918	<0.001^∗^			
IIB	99.073	12.857 to 763.431	<0.001^∗^			
IIIA	142.128	19.164 to 1054.090	<0.001^∗^			
P53 expression			<0.001^∗^			
Negative	Reference					
Positive	5.217	2.577 to 10.564				
Ki-67 expression			<0.001^∗^			0.025^∗^
<10%	Reference			Reference		
≥10%	8.412	3.632 to 19.486		2.954	1.146 to 7.614	
EGFR mutation			0.083			
Wild type	Reference					
Mutation	2.336	0.895 to 6.098				

IQR: interquartile range; CEA: carcinoembryonic antigen; AIS: adenocarcinoma *in situ*; MIA: minimally invasive adenocarcinoma; IAC: invasive adenocarcinoma; SCC: squamous cell lung cancer; EGFR: epidermal growth factor receptor. ^∗^*P* < 0.05.

**Table 3:** Point assignment, prognostic scores, and estimated 5-year overall survival based on pathological tumor size, lymph node metastasis, and Ki-67 expression.

Variable	Prognostic score	Estimated 5-year overall survival (%)
Pathological tumor size (mm)		
≤10 mm	10	
10-20 mm	5	
>20-30 mm	0	
Lymph node metastasis		
N0	4.72	
N1	2.36	
N2	0	
Ki-67 protein expression		
<10%	7.95	
≥10%	0	
Total prognostic score (IQR)		
0-9.72		57.8
9.72-17.67		85.3
7.67-22.67		97.2
≥22.67		100

IQR: interquartile range.

**Table 4:** Kaplan-Meier survival analysis of nomogram score groups for the training and validation sets.

Group	0-9.72 (*P* value)	9.72-17.67 (*P* value)	17.67-22.67 (*P* value)	≥22.67 (*P* value)
Training set				
0-9.72	—	0.022	<0.001	<0.001
9.72-17.67	0.022	—	0.007	0.001
17.67-22.67	<0.001	0.007	—	0.569
≥22.67	<0.001	0.001	0.569	—
Validation set				
0-9.72	—	0.067	0.013	0.001
9.72-17.67	0.067	—	0.106	0.072
17.67-22.67	0.013	0.106	—	0.512
≥22.67	0.001	0.072	0.512	—

## Data Availability

The data used to support the findings of this study are available from the corresponding authors upon request.
